# First Detection of *Listeria monocytogenes* in a Buffalo Aborted Foetus in Campania Region (Southern Italy)

**DOI:** 10.3389/fvets.2020.571654

**Published:** 2021-02-10

**Authors:** Claudia Esposito, Lorena Cardillo, Giorgia Borriello, Grazia Ascione, Ornella Valvini, Giorgio Galiero, Giovanna Fusco

**Affiliations:** ^1^Unit of Virology, Department of Animal Health, Istituto Zooprofilattico Sperimentale del Mezzogiorno, Naples, Italy; ^2^Unit of Applied Biotechnologies and Bioinformatics, Department of Animal Health, Istituto Zooprofilattico Sperimentale del Mezzogiorno, Naples, Italy; ^3^Unit of Special Diagnostics and Fish Pathology, Department of Animal Health, Istituto Zooprofilattico Sperimentale del Mezzogiorno, Naples, Italy; ^4^Istituto Zooprofilattico Sperimentale del Mezzogiorno, Scientific Director, Naples, Italy

**Keywords:** *Listeria monocytogenes* (LM), serogroup IVb, water buffalo (*Bubalus bubalis*), abortion-spontaneous, bubaline foetus

## Abstract

*Listeria monocytogenes* (LM) is the causative agent of listeriosis in both animals and humans, representing one of the most severe food-borne diseases in humans. Out of 13 serotypes, only three (i.e., 1/2a, 1/2b, and 4b) are responsible for 95% of human outbreaks of listeriosis. Ruminants have been hypothesised to represent the main natural reservoir for this pathogen and to be involved in the transmission of *Listeria* to humans. During pregnancy, listeriosis in ruminants cause various reproductive disorders as well as abortion. However, little is known about abortion due to LM in water buffaloes (*Bubalus bubalis*). In this study, we report for the first time the detection of LM in a water buffalo foetus in the region of Campania, Italy. Complete necropsy was performed, and samples and swabs from the abomasum, kidneys, liver, lungs, and spleen were collected. Microbiological and molecular analyses were carried out to detect bacterial, viral, and protozoarian abortive pathogens. The results revealed the presence of LM in the liver, lungs, and abomasum, and no other agent was detected. Isolation was confirmed by biochemical and molecular tests. Molecular serotype characterisation was performed, and serogroup IVb was identified. In conclusion, because of the zoonotic implications of our findings, this report highlights the importance of including LM in the diagnostic panel in cases of bubaline abortion.

## Introduction

*Listeria monocytogenes* (LM) is a food-borne pathogen that causes listeriosis, a zoonotic disease that affects several animal species, including humans. In humans, listeriosis is considered to be a potentially lethal disease, especially in immunocompromised individuals. Notably, this disease is characterised by septicaemia, meningitis, and, more frequently, meningoencephalitis and abortion. Several gastro-intestinal manifestations with fever can also occur (1, 2).

Generally, LM is a ubiquitous, rod-shaped, Gram-positive bacterium that belongs to family Listeriaceae. In total, 20 species of *Listeria* are currently known (3), with LM and *L. ivanovii* being the only pathogenic species. While *L. ivanovii* are rarely involved in human cases of infection, LM have been recognised as the main species involved in food-borne outbreaks in both animals and humans (2, 4).

In nature, LM strains differ in terms of their epidemiological potential and their ability to cause disease in humans (5). However, several studies have revealed a genetic variation in the isolated populations. This variability is believed to be strongly related to both the microbial load necessary to induce infection and membership to a particular lineage (6, 7). Generally, LM consists of four different lineages (i.e., I, II, III, and IV), with different but overlapping ecological niches, that include 13 serotypes ranging from low to high virulence (1/2a, 1/2b, 1/2c, 3a, 3b, 3c, 4a, 4ab, 4b, 4c, 4d, 4e, and 7) (1, 8). Most LM isolates belong to lineages I and II, whose serotypes 1/2a (lineage II) and 1/2b and 4b (lineage I) are associated with human outbreaks (8–10), although strains from lineages III and IV are occasionally associated with human diseases (1, 11, 12).

The main transmission route of this disease is believed to be via the consumption of contaminated food and raw milk. The environment in which food processing occurs is considered an important source of infection for humans (4).

In animals, LM has been isolated from several species, including mammals, birds, and fish (1, 13–15). Ruminants are considered to be the most susceptible species (2) and are believed to be the main reservoir for human infection (16). The main route of infection is believed to be via contaminated feedstuff. Once ingested, the bacterium reaches the target organs (i.e., liver, spleen, brain, and uterus) via the lympho-hematogenous pathway (17). Notably, this disease can be asymptomatic or can present with septicemia and lesions mainly in the central nervous system (meningitis, meningoencephalitis, and rhombencephalitis) (16, 18). Notably, mastitis due to LM is quite rare. However, isolation of the bacterium from raw milk can prove the onset of specific mammary infection (2, 4, 19). LM is also associated with various reproductive disorders, fetal infection, placentitis, and spontaneous abortion, especially during the last trimester of pregnancy (20). Abortion due to LM is widely described in cattle and small ruminants (20, 21), whereas little is known about this condition in water buffalos (*Bubalus bubalis*). Notably, LM has been isolated from meat and milk samples collected from buffaloes (22), and low prevalence has been found in the uterus and in animals with reproductive disorders (23–25).

In this report, we describe the detection and isolation of LM in the liver, abomasum, and lungs of a water buffalo foetus. Molecular characterisation revealed that the isolated strain belonged to serogroup IVb, which includes serotypes 4b, 4d, and 4e. To the best of our knowledge, this is the first report of the detection and isolation of LM in a bubaline aborted foetus in Southern Italy.

## Methods

### Anatomopathological and Microbiological Examinations

A bubaline foetus was first submitted to a complete necropsy examination, which was performed within 24 h after death, by postgraduate veterinarians from Istituto Zooprofilattico Sperimentale del Mezzogiorno (IZSM), Portici, Italy, according to standard protocols (26). Surface cauterisation was performed, and organs were incised using sterile scalpels. Approximately 2 cm^2^ samples and swabs were collected from the inner parts of the organs. Specimens were processed for bacteriological analysis within 2 h.

Nucleic acid extraction was conducted as follows: 2 mg of tissue samples was first suspended in 2 ml of sterile phosphate-buffered saline in 2 ml tubes, homogenised with glass beads using a TissueLyser (Qiagen, Hilden, Germany) and centrifuged for 5 min at 1,700 × g. Aliquots of 200 μl of the supernatant were then collected, and nucleic acid extraction was performed using a QIAsymphony automated system (Qiagen), processed according to the manufacturer's protocol, eluted in 60 μl, and stored at −80°C until use. All viral, bacterial, and protozoarian abortive pathogens were investigated (Table 1).

**Table 1:** Diagnostic protocols of the abortive pathogens.

**Pathogen**	**Matrix**	**Test**	**References**
Bovine herpesvirus 1 (BHV-1)	Lungs, liver	PCR	(27)
Bovine herpesvirus 4 (BHV-4)	Lungs, spleen	PCR	(28)
Bovine viral diarrhea virus (BVDV)	Spleen	PCR	(29)
*Brucella* spp.	Abomasum content, liver, lungs, spleen	Bacteriological	(30)
*Campylobacter fetus*	Abomasum content, liver	Bacteriological, PCR	(31)
*Chlamydophila abortus*	Liver, lungs, abomasum content	PCR	(28)
*Coxiella burnetii*	Liver, lungs, abomasum content	PCR	(28)
*Listeria* spp.	Abomasum content, liver, lungs	Bacteriological, PCR	(15, 32, 33)
*Neospora caninum*	Heart	PCR	(28)
*Toxoplasma gondii*	Heart	PCR	(28)
*Leptospira* spp.	Kidney	PCR	(28)
*Salmonella* spp.	Liver	Bacteriological	(34)

Notably, LM was determined according to the procedures described by the World Organisation for Animal Health (OIE) in the Manual of Diagnostic Tests and Vaccines for Terrestrial Animals (15). Swabs were plated in a primary selective enrichment broth, Half Fraser Broth (Oxoid, Rodano, Italy), followed by secondary enrichment in Half Fraser Broth, both incubated at 30 ± 1°C for 24 h. Culture broth was inoculated on selective Oxford Agar (Oxoid) in duplicate and incubated for 24–48 h at 37 ± 1°C. Five colonies, presumed to be *Listeria* spp., were isolated and picked for confirmation. Each colony was plated on Tryptone Soya Yeast Extract Agar (Oxoid) and incubated at 37 ± 1°C for 24 h. The colonies were then submitted to identification on the basis of biochemical macro-methods, Gram stain, catalase, and oxidase tests and hemolysis on Blood Agar (Oxoid), as well as miniaturised biochemical procedures with VITEK 2 Compact (bioMérieux, Lyon, France).

### LM Serogrouping by PCR

Bacterial colonies were grown on Ottaviani and Agosti agar (ALOA agar; Oxoid) at 37°C for 24 h. Typical LM colonies (blue-green colour with an opaque halo) were collected for DNA extraction using an InstaGene Matrix (Bio-Rad Laboratories, Hercules, CA, USA) following the manufacturer's instructions. Molecular serogrouping was performed using multiplex PCR by simultaneously amplifying *PrfA, lmo0737, lmo1118, ORF2819*, and *ORF2110* serotype-specific marker genes as well as the *prs* gene, specific for strains of the genus *Listeria* (Table 2) (32, 33). The PCR reaction was performed in a final volume of 25 μl and included 2 μl of template, with a DNA concentration of 25 ng/μl and HotStarTaq PCR Master Mix 1X (Promega, Madison, WI, USA), along with MgCl_2_ (final concentration: 2 mM); primers LMO1118-1 and LMO1118-2 (0.8 μM); primers LMO0737-1, LMO0737-2, ORF2110-1, ORF2110-2, ORF2819-1, and ORF2819-2 (0.4 μM); primers LIP1 and LIP2 (0.2 μM); and primers PRS1 and PRS2 (0.1 μM). The thermal profile consisted of one cycle at 95°C for 15 min for Taq activation and then 35 cycles at 94°C for 30 s, 53°C for 40 s, and 72°C for 1.5 min, followed by a last extension cycle at 72°C for 7 min. Each working session also included the following positive control samples [reported using the Administración Nacional de la Seguridad Social (ANSES) reference DNA number]: 00EB248LM (serogroup IIa), 00EB249LM (serogroup IIb), 00EB250LM (serogroup IIc), 00EB254LM (serogroup IVa), 00EB256LM (serogroup IVb), and *Listeria ivanovii* ATCC19119 (serogroup L).

**Table 2:** Primer sets for molecular serogrouping of *Listeria monocytogenes*.

**Gene target**	**Nucleotide sequence**
*PrfA*	LIP1 5′ -GATACAGAAACATCGGTTGGC-3′
	LIP2 5′ -GTGTAATCTTGATGCCATCAGG-3′
*Prs*	PRS1 5′ -GCTGAAGAGATTGCGAAAGAAG-3′
	PRS2 5′ -CAAAGAAACCTTGGATTTGCGG-3′
*Lmo0737*	LMO0737-1 5′ -AGGGCTTCAAGGACTTACCC3′
	Lmo0737-2 5′ -ACGATTTCTGCTTGCCATTC-3′
*Lmo1118*	LMO1118-1 5′ -AGGGGTCTTAAATCCTGGAA-3′
	Lmo1118-2 5′ -CGGCTTGTTCGGCATACTTA-3′
*orf 2819*	ORF2819-1 5′ -AGCAAAATGCCAAAACTCGT-3′
	ORF2819-2 5′ -CATCACTAAAGCCTCCCATTG-3′
*orf2110*	ORF2110-1 5′ -AGTGGACAATTGATTGGTGAA-3′
	ORF2110-2 5′ -CATCCATCCCTTACTTTGGAC-3′

## Results

In December 2018, during a routine analysis, a bubaline foetus was brought from a farm located in the province of Caserta, Campania region, Southern Italy, and presented to IZSM in Portici, Naples, Italy, to investigate the cause of abortion. Anamnesis showed that the abortion occurred in the last third of gestation with no prodromal symptoms. A brucellosis eradication campaign, based on serum agglutination and complement fixation tests (30), was performed on the farm in July of the same year, and negative results were obtained for the mother of the aborted foetus.

The foetus was submitted to complete necropsy examination. Organs were *in situ* and normal in shape and size. Severe abdominal and moderate pleural serohematic effusions were observed, and mild pericardial serohematic fluid was found when the pericardium was opened.

**Figure 1:**
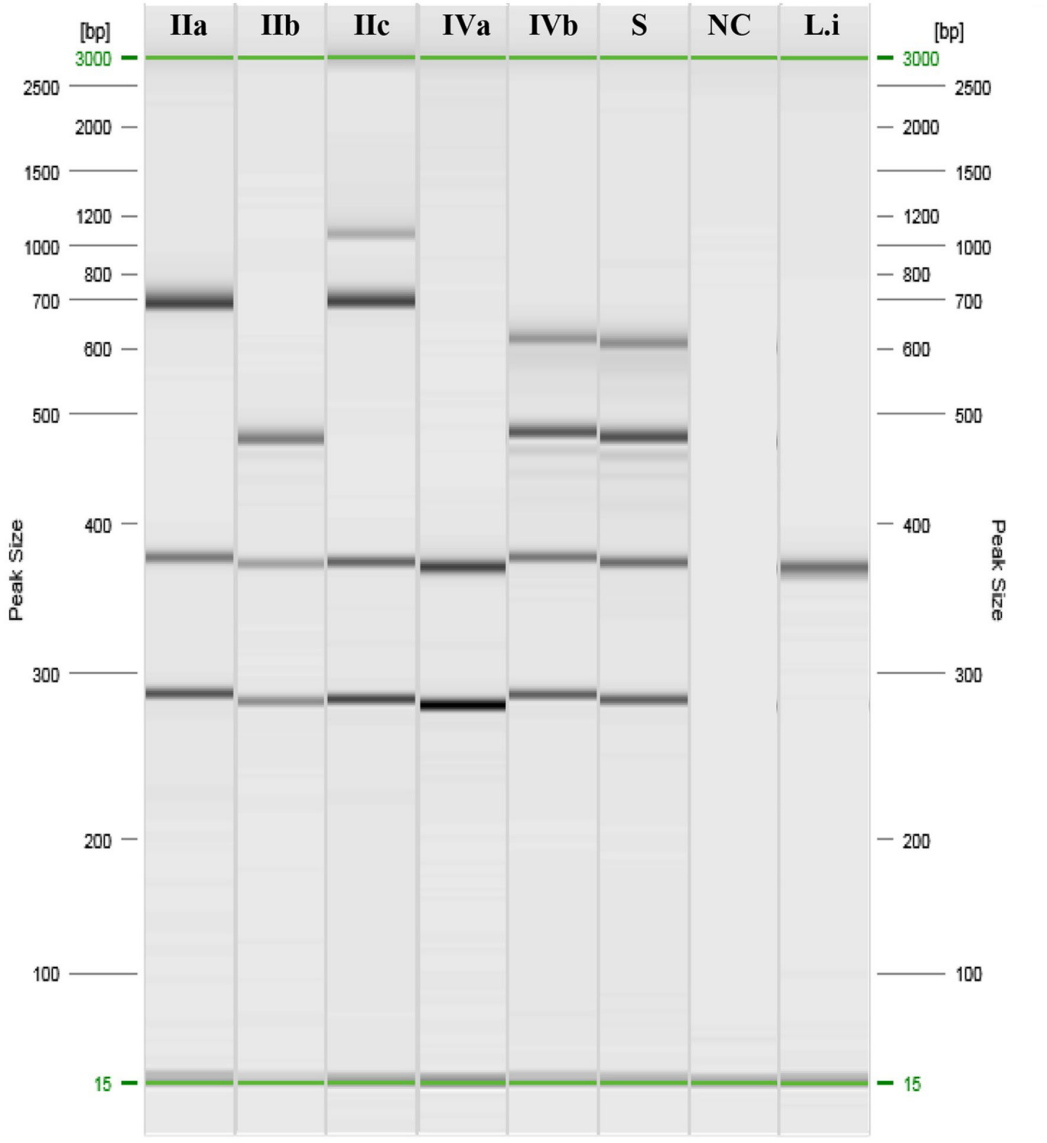
PCR results of *Listeria monocytogenes* serogrouping. IIa: ref. Anses 00EB248LM; IIb: ref. Anses 00EB249LM; IIc: ref. Anses 00EB250LM; IVa: ref. Anses 00EB254LM; IVb: ref. 00EB256LM; S, Sample; NC, Negative control; Li, *Listeria ivanovii* ATCC19119.

Samples and swabs were obtained from the lungs, liver, spleen, abomasum, heart, and kidneys and tested for abortive viruses, bacteria, and protozoa (Table 1). Notably, LM was isolated on Oxford Agar from the liver, lungs, and abomasum swabs, and no other agent was detected. The isolated colonies were then submitted for biochemical confirmation using macro-methods and miniaturised procedures (VITEK 2 Compact; bioMérieux). The isolates were then analysed to determine the molecular serogroup. PCR results (Figure 1) indicated that this strain could be classified as LM serogroup IVb (corresponding to serotypes 4b, 4d, and 4e).

## Discussion

LM is considered one of the most insidious food-borne pathogens that primarily affects individuals at high risk, such as immunocompromised patients, pregnant women, elderly people, and young children, resulting in a mortality rate of around 15–20% (35–37). Human infection occurs as a result of the consumption of contaminated food items, such as milk and dairy products, seafood, and food of animal origin (38). Notably, LM can cross the placental barrier and infect the foetus, causing spontaneous abortion, stillbirth, and even pre-term and neonatal infections in both animals (39) and humans (40). In this study, we report for the first time the detection of LM in a water buffalo foetus that got aborted in December 2019 in the region of Campania, Italy. As described in both cattle and small ruminants, abortion occurred during the last period of gestation (20, 21) and no gross lesions were observed (15, 41).

In ruminants, abortions due to *Listeria* are characterised by a seasonal trend, as they occur mostly in winter or early spring as a result of the higher consumption of contaminated silage, which is recognised as the main route of transmission in these species (20, 21, 42). Furthermore, under certain cold climatic conditions, LM can show a higher pathogenic profile than heat seasons (43).

In humans, from the 13 identified LM serotypes, serotypes 1/2a, 1/2b, and 4d have been identified to cause disease in more than 95% of the cases (44, 45). While serotypes 1/2a and 1/2b have been recognised to cause sporadic illness, serotype 4b is believed to be the main cause of human outbreaks (1, 44). It has been speculated that clones of serotype 4b are more virulent than other strains, although this has not yet been reproduced in infection models (1). Molecular serotyping of LM strains allows the determination of five distinct molecular groups correlated with the serotypes, which allow the correct classification of the most common disease-associated serotypes (i.e., 1/2a, 1/2b, 1/2c, 4a, and 4b) into unique serogroups (33). Notably, the clinical isolates characterised in the present study belong to serogroup IVb, which includes serotypes 4b, 4d, and 4e. This group has been found in several clinical cases from both humans and different farm animal species, such as cattle, sheep, poultry and swine, in different countries (46–48). Serogroup IVb has also been found to be the predominant group among cases of listeriosis in small ruminants in Greece (49), accounting for 68% of all clinical isolates from encephalitis cases and 57% of the isolates from milk samples. Similarly, in Italy, serogroup IVb has been found to be the most represented among rhombencephalitis-*Listeria*-associated strains from cattle (16). In buffaloes, LM was isolated from meat and milk (22) and sporadic infections in animals with reproductive disorders (23–25), but none of the serotypes has been investigated. However, no abortion due to LM has yet been described in water buffaloes.

The connection between ruminant and human listeriosis is still not clearly understood, as direct transmission between these two species rarely occurs (50). However, it is believed that ruminants act as a reservoir for LM (16). Notably, LM can contaminate food through feces and water (50), and both diseased and asymptomatic animals can discharge LM in their milk and feces, making meat and milk a real risk factor for human infection (21, 51).

In conclusion, considering the zoonotic role of LM and the significance of the findings of this report, it can be concluded that LM serogroup IVb plays a crucial role as a potential abortive agent in water buffaloes and should, therefore, be considered in the diagnostic panel in cases of bubaline abortion.

## Data Availability Statement

All datasets presented in this study are included in the article/supplementary material.

## Ethics Statement

Ethical review and approval was not required for the animal study because the experimental Zooprophylactic institutes (IIZZSSMM) are official laboratories designed by the Italian Ministry of Health that are involved in epidemiological surveillance, animal health, food, and feed safety and diagnostics. For this reason, ethics approval was deemed unnecessary in agreement with institutional policy and national regulations. Written informed consent for participation was not obtained from the owners because the study was conducted during institutional activities.

## Author Contributions

All the authors equally contributed to the study. CE, LC, GB, GA, and OV drafted the manuscript. GG and GF conceded and revised the study. OV and GB conducted the microbiological and biotechnological exams.

## Conflict of Interest

The authors declare that the research was conducted in the absence of any commercial or financial relationships that could be construed as a potential conflict of interest.
